# Accelerated Adaptive Laboratory Evolution by Automated Repeated Batch Processes in Parallelized Bioreactors

**DOI:** 10.3390/microorganisms11020275

**Published:** 2023-01-20

**Authors:** Lukas Bromig, Dirk Weuster-Botz

**Affiliations:** Chair of Biochemical Engineering, Technical University of Munich, Boltzmannstraße 15, D-85748 Garching, Germany

**Keywords:** adaptive laboratory evolution (ALE), *Escherichia coli*, process development, repeated batch process, process automation, growth rate optimization, glycerol utilization, biomass estimator, black box model, batch process

## Abstract

Adaptive laboratory evolution (ALE) is a valuable complementary tool for modern strain development. Insights from ALE experiments enable the improvement of microbial cell factories regarding the growth rate and substrate utilization, among others. Most ALE experiments are conducted by serial passaging, a method that involves large amounts of repetitive manual labor and comes with inherent experimental design flaws. The acquisition of meaningful and reliable process data is a burdensome task and is often undervalued and neglected, but also unfeasible in shake flask experiments due to technical limitations. Some of these limitations are alleviated by emerging automated ALE methods on the μL and mL scale. A novel approach to conducting ALE experiments is described that is faster and more efficient than previously used methods. The conventional shake flask approach was translated to a parallelized, L scale stirred-tank bioreactor system that runs controlled, automated, repeated batch processes. The method was validated with a growth optimization experiment of *E. coli* K-12 MG1655 grown with glycerol minimal media as a benchmark. Off-gas analysis enables the continuous estimation of the biomass concentration and growth rate using a black-box model based on first principles (soft sensor). The proposed method led to the same stable growth rates of *E. coli* with the non-native carbon source glycerol 9.4 times faster than the traditional manual approach with serial passaging in uncontrolled shake flasks and 3.6 times faster than an automated approach on the mL scale. Furthermore, it is shown that the cumulative number of cell divisions (CCD) alone is not a suitable timescale for measuring and comparing evolutionary progress.

## 1. Introduction

Adaptive laboratory evolution (ALE) is increasingly gaining relevance in industrial biotechnology as a tool for improving production strains [1]. Insights gained by adaptive laboratory evolution through whole-genome sequencing can be directly applied to the fields of metabolic engineering (reverse engineering) and complemented with classical genetic engineering to yield improved microbial production systems [2,3,4,5,6]. Most publications focus on the evolutionary aspects or applications of ALE, which have been reviewed extensively [1,7,8,9]. The techniques and experimental setups are increasing in their degree of automation and sophistication as shown by Radek et al. (2017), who translated ALE to an automated microtiter plate format, or Wang et al. (2020), who used a microbial microdroplet culture platform [10,11,12,13]. Wong et al. (2018) and Ekkers et al. (2020) described a low-cost automated batch system on the mL scale and LaCroix et al. (2017) used an automated batch system on the mL scale for ALE growth experiments of *E. coli* on glycerol [14,15,16]. However, automation efforts have so far been focused on miniaturization and parallelization but not on optimization, process control, and data quality. ALE methods for chemostat cultures have a different field of application and were not considered in this study [10,12]. The serial passaging of batch cultures, a tedious and labor-intensive task, is the most popular ALE method [8]. However, the way serial passaging is performed brings several disadvantages that impact the outcome of ALE experiments and are highly inefficient.

Designing the right stress for the sought after target is a crucial part of ALE experiment design. Studies that aim to improve the microbial growth rate or growth with non-native carbon sources rely on the serial passaging of batch experiments. A major driver for improving growth rates of microbial cell factories with alternate carbon sources is the increasing market pressure to utilize surpluses or cheaper substrates in bioprocesses that produce fine and bulk chemicals that compete with classical chemical processes [17] and the general aim to improve space–time yields. In these cases, the specific growth rate is used as the metric of fitness since the efficiency of carbon source utilization is rate-limiting and directly coupled to growth [8,18]. Propagation during the exponential phase and a reduction in or even elimination of a lag phase become important as a prolonged stationary phase or lag phase may shift the evolutionary pull towards an undesired fitness archetype, thus allowing other traits to be selected for [8,16,19,20].

Furthermore, genetic evolution is sensitive to the process conditions; thus, state variables must be controlled tightly [21]. Process control, the norm in liter scale stirred-tank bioreactors, is limited in shake flask cultures. The classical manual approach of serial passaging in shake flasks results in a suboptimal stress design that has become accepted in the scientific community. Comparable automated ALE batch experiments operating on the mL scale have limited capabilities for process control, i.e., a lack of pH and DO control, as well as the continuous data acquisition of key state variables [14,16]. The dynamic nature of batch cultivations results in specific growth rates, cell densities, and environmental conditions that change continuously not only during a single batch but, inherently in ALE processes specifically, also between each sequential batch. This extra level of dynamics has one major drawback: the process time of the batches decreases over time and the process time can only be estimated from the results of the previous batch. In order to maintain a consistent schedule, individual batches are artificially prolonged to fit a predefined, usually daily, passaging schedule by adjusting the initial cell concentration to fit a forecasted 24 h process. However, this results in at least one of the following problems: (1) the initial cell concentration can affect the duration of the lag phase [22,23,24] and a very small passage size may lead to beneficial mutations being lost [16]. Hence, the initial cell concentration should be large enough and kept constant between batches, which also results in lag-phase minimization, which is beneficial for ALE experiments targeting the growth rate and growth on non-native carbon sources. (2) The cells enter the stationary phase for an unknown duration before the passaging, making the calculation of the specific growth rate for this batch unreliable and inducing the wrong selection stress as the organism is cultivated in growth-limiting conditions. Lee et al. suggested the cumulative number of cell divisions (CCD) as a meaningful parameter for measuring the evolutionary speed in ALE experiments and that fundamental kinetics can be derived [25]. However, deriving transferable insights from such a multivariate experiment is complex and using CCD as such a metric has multiple limitations. The CCD does not take into account the number of performed passages, the passage size, or other factors that depend on the reactor system, such as the reactor volume.

In this study, a comparative analysis was performed in which experiments from Lee et al. (2011) were reproduced in the proposed automated ALE experimental setup. The results were compared with experimental data from manual and automated systems [2,16,25,26]. It is further shown that an alternative experimental setup can greatly improve the efficiency at which these experiments are conducted, not just regarding the timescale of evolution but also by reducing the impact of undesired stresses caused by prolonged stationary and lag phases or large temporal changes in initial biomass concentrations.

## 2. Material and Methods

### 2.1. Bacterial Strain

The experiments were carried out using fresh cultures of the wild-type strain *E. coli* K12 MG1655 from the German Collection of Microorganisms and Cell Cultures (#DSM 18039, DSMZ GmbH, Braunschweig, Germany). The same wild-type strain was used in the reference literature [2,25,26]. The freeze-dried culture was prepared in LB medium according to DSMZ instructions and stored as cryo-stock at −80 °C in 50% glycerol.

### 2.2. Media

Seed cultures were grown with lysogeny broth (LB) medium containing, per liter, 10 g peptone, 5 g yeast extract, and 5 g NaCl, which was adjusted to pH 7 with NaOH. The repeated batch processes were conducted with medium according to Riesenberg et al. (1991) (RB) containing, per liter, 8.4 mg EDTA, 8.4 mg CoCl_2_·6H_2_O, 15 mg MnCl_2_·4H_2_O, 1.5 mg CuCl_2_·2H_2_O, 3 mg H_3_BO_3_, 2.5 mg Na_2_MoO_4_·2H_2_O, 13 mg Zn(CH_3_COO)_2_·2H_2_O, 0.1 g Fe(III) citrate, 13.3 g KH_2_PO_4_, 4 g (NH_4_)_2_HPO_4_, 1.7 g citric acid·H_2_O, 2.4 g NaOH, 1.2 g MgSO_4_·7H_2_O, and the carbon source glycerol at a concentration of 15 $g\;L^{-1}$ [27]. Anti-Foam (AF 204, Sigma-Aldrich, Merck KGaA, Darmstadt, Germany) was added at a concentration of 1 mL L${}^{-1}$. The RB medium used in experiments with mutagen contained an additional 5 mg L${}^{-1}$ of 1-methyl-3-nitro-1-nitrosoguanidine (NTG, CAS-#: 70-25-7, Biosynth s.r.o, Bratislava, Slovak Republic). All media were sterilized in an autoclave at 120 °C for 20 min. NTG was sterile-filtered and added to the media flasks just prior to process start. A list with additional information on used chemicals and media preparation is provided in the Supplementary Materials.

### 2.3. Seed Culture

A total of 0.1 mL inoculum (cryo-stock) was added to 100 mL of autoclaved LB medium in baffled 500 mL Erlenmeyer flasks. Seed cultures were incubated in a rotary shaker (Multitron, Infors AG, Bottmingen, Switzerland) at 37 °C and 150 rpm up to 12 h until a final cell concentration of $OD_{600}$ of 2–2.5 was reached. If necessary, cell suspensions were concentrated by an additional centrifugation and resuspension step to obtain the required inoculum concentration.

### 2.4. Experimental Setup and Process Control

Batch cultivations were performed in 4 parallel stirred-tank bioreactors (DASGIP Parallel Bioreactor System, Eppendorf AG, Hamburg, Germany) on an L scale, each with a total working volume of 575 mL. Autoclaved RB medium, including the carbon source glycerol, was used for the repeated batch processes to enable unlimited growth conditions. The proposed process achieves higher cell densities and, to avoid limitations, RB medium with a higher nitrogen, sulfur and phosphorus content, compared to the M9 medium used in related works [2,16,25,26], was used. The composition of the RB medium used in this study is closely related to the M9 medium used in the reference cultivations. Kangwa et al. (2015) showed that both synthetic, mineral media (RB, M9) result in the same biomass production and, thus, that the impact on growth rate is expected to be negligible [28]. The initial inoculation was performed manually to yield an initial biomass concentration of 0.2 g L${}^{-1}$ in the individual stirred-tank bioreactors, whereas the inoculation of the sequential batches was performed indirectly as described in the following section about the reactor setup, resulting in measured initial biomass concentrations between 0.15–0.4 g L${}^{-1}$. The pH was controlled using 12.5% (v/v) NH_4_OH and 2 M HCl, which were added via the peristaltic pumps of the reactor system to maintain pH 7. The bioreactor was aerated with an air flow set between 1.16–3.48 vvm. Processes were started with a stirrer rate of 600 rpm and regulated up to speeds of 1200 rpm. Dissolved oxygen (DO) levels and pH were measured continuously using a DO probe (Visiferm DO ECS 225 H0, Hamilton Bonaduz AG, Bonaduz, Switzerland) and a pH probe (EasyFerm Plus PHI K8 225, Hamilton Bonaduz AG, Bonaduz, Switzerland). The pH probe was calibrated before autoclaving by 2-point calibration and the DO probe was calibrated before process start by first aerating the medium with pure nitrogen (DO =0%) followed by aeration with pressured process air until equilibrated (DO =100%). The reactors were equipped with a PT-100 temperature probe (Platinum RTD temperature sensor, Eppendorf AG, Hamburg, Germany) and a level sensor (Level Sensor, Eppendorf AG, Hamburg, Germany). Off-gas was condensed and analyzed for on-line measurement of CO_2_ and O_2_ (BlueVary, BlueSens gas sensor GmbH, Herten, Germany).

**Figure 1:**
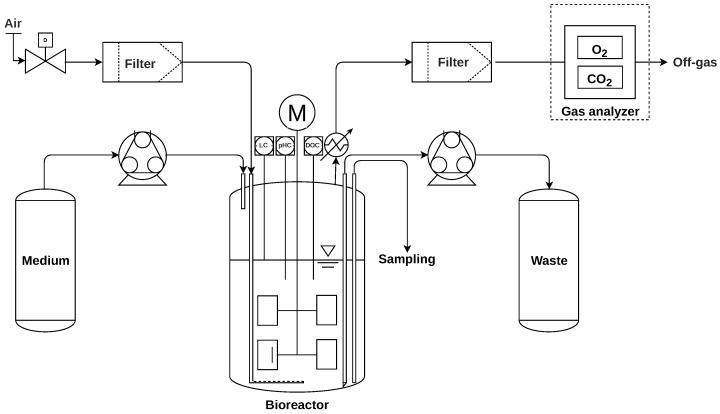
A simplified scheme of the experimental setup with one bioreactor is shown. Experiments were performed in four parallelized stirred-tank bioreactors. Sterile, pressured air was sparged at the bottom of the bioreactor and, upon exit, condensed at the bioreactor gas outlet by a reflux condenser, subsequently sterile-filtered, and passed through the off-gas analyzer. The RB medium was provisioned in 5 L flasks, which were exchanged with fresh medium every eighth batch. Removed biosuspension was pumped into a 50 L shared waste container. The bioreactor was filled and emptied by peristaltic pumps. A rising pipe with a beveled needle point style end (bevel angle of 90°) was constructed to empty the bioreactor adequately. In-line probes for medium exchange-control, pH-control, and DO-control were inserted through the bioreactor head. Two Rushton turbines (Rushton-Type stirrer, Eppendorf, Hamburg, Germany) were placed on the stirrer axis starting 5 mm above the end of the axis, with a vertical distance of 25 mm between the turbine wings.

ALE experiments were performed by passaging in a repeated batch mode for up to 200 h. The experimental setup is shown in Figure 1. Experiments were conducted with glycerol as a carbon source with and without the mutagen NTG to create experiments comparable to the ones presented by Fong et al. (2005), Herring et al. (2006), Lee et al. (2011), and LaCroix et al. (2017) [2,16,25,26] and to validate the acceleration potential of the proposed setup to a manual and another automated method. Cultivations were performed at 37 °C, the same temperature at which the experiments of Lee et al. (2011) were executed at. In contrast, the set of experiments conducted by Fong et al. (2005), which were used by Herring et al.(2006), were performed at 30 °C. LaCroix et al. (2017) did not specify a cultivation temperature in their publication or Supplementary Materials. These limitations must be considered when directly comparing the presented results with the data derived from the experiments of Fong et al. (2005) and LaCroix et al. (2017). ALE experiments were stopped manually once a stable phenotype, i.e., the specific growth rate, was detected in at least three consecutive batches by photometric at-line measurements. Samples of the last evolution population were taken, diluted with 50% glycerol, and stored in a freezer at −80 °C.

#### 2.4.1. Off-Gas Analysis

The concentrations of CO_2_ and O_2_ were analyzed using an external off-gas analyzer (BlueVary, BlueSens gas sensor GmbH, Herten, Germany), which was integrated into the external process control software using a driver that was developed as part of this work according to the Standardization in Laboratory Automation 2 device interface standard (SiLA 2).

#### 2.4.2. Peristaltic Pumps

Two peristaltic pumps with four individually controllable channels (Ismatec Reglo ICC, Cole- Parmer, Wertheim, Germany) were used to pump out the biosuspension and refill the bioreactors with fresh medium. Thus, each bioreactor required two individually controllable pump channels. The pumps were integrated and controlled by the external process control software via a SiLA 2 driver developed for this pump as part of this work.

#### 2.4.3. Process Control Software

The proprietary process control software (DASware^®^ Control, Eppendorf AG, Hamburg, Germany) was used for the basic batch process control, such as pH control, DO control, and temperature control. Process control logic related to ALE and passaging was performed using the workflow and scripting environment of an external control software (SiLA 2 Manager) that was developed in previous works [29]. To enable the integration of the reactor system into an external control software, the proprietary vendor software DASWare^®^ Connect is required, which provides an OPC-UA interface. As part of this work, a driver was developed that translates this interface into a standardized interface following the Standardization in Laboratory Automation specification (SiLA 2, Rapperswil-Jona, Switzerland). The integration of all devices in the external control software via the standardized interfaces enabled the acquisition of all relevant process data in a central database (InfluxDB, InfluxData Inc., San Francisco, CA, USA). Process data of the bioreactor system and the off-gas analyzer were acquired in 15 *s* intervals. A control script, written in the external control software’s Python editor (Supplementary Materials), orchestrated the passaging process between the batch phases by accessing and controlling the bioreactor system as well as the peristaltic pumps. All drivers were written in Python using the SiLA 2 Python reference implementation [30].

#### 2.4.4. Repeated Batch Mode

**Figure 2:**
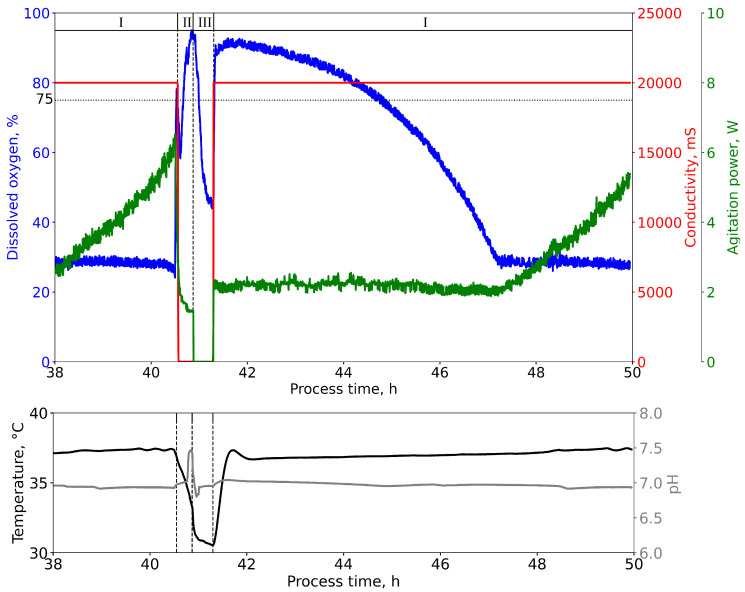
Process data of the transition between two consecutive batches is shown and classified into three distinct phases: the batch phase (I), the draining phase (II), and the refill process phase (III). The transition from phase (I) to (II) is triggered by a DO signal that surpasses a set threshold of 75 % air saturation. The duration of the draining process is dependent on the maximum calibrated pump rate of the respective channel. The pump is addressed with the pump rate by volume mode and parametrized with the bioreactor volume, including an additional buffer volume to ensure complete removal of the biosuspension. The transition from phase (II) to (III) is triggered by a rise in the conductivity signal over a fixed threshold of 200 mS. Agitation is automatically down-regulated during phase (II) and switched off during phase (III).

Figure 2 shows exemplary process data to explain the applied control scheme of the passaging process. Passaging is triggered automatically at the end of the exponential growth phase (I), which is detected by a sudden spike in DO due to depletion of the carbon source (glycerol). The pump action is triggered if a DO signal threshold of 75% air saturation is surpassed. In order to avoid faulty executions during the early stages of the batch process in which a DO >75% is to be expected, a minimal batch time of 1 h was defined, during which, this mechanism is deactivated. The peristaltic pumps in Figure 1 are connected to the bioreactor head via reliable, sterile quick-couplings (PMCD220212 and PMCD170212, Colder Products Company, Mörfleden-Walldorf, Germany). Five-liter glass flasks (DU218017304, DWK Life Sciences GmbH, Wertheim Germany) were used for media storage, which had to be replaced every eighth batch. A rising pipe with a sharp phased-off end was confectionized for the waste stream so that the bioreactor could be emptied almost entirely. Temperature and pH control were switched off during the transfer process (II + III, Figure 2) to avoid erroneous controller behavior after dry running of the sensors. Agitation and aeration were continued during the draining process (II). During the refill process (III), agitation was stopped, whereas the aeration was continued to ensure a sterile environment and minimal oxygen availability. The bioreactor level sensors were calibrated to a working volume of 575 mL before the start of the ALE experiment and provided the input for the stop trigger of the peristaltic pumps during the refill process. As shown in Figure 2, phase III, the pumping action was stopped once a conductivity of greater than 200 mS was detected by the level sensor, which translates to the earliest contact between medium and probe. Each bioreactor was controlled separately.

### 2.5. Specific Growth Rate Estimation Using a Black-Box Model

#### 2.5.1. Black Box Model

To describe the change in biomass and substrate concentration over the course of the ALE experiment and to derive process parameters such as specific growth rate and CCD, the captured on-line data were used as input for a black-box model. The conversion rates ($r_{i}$ in mol of i/h) are derived from a simplified metabolic black-box equation for aerobic *E. coli* growth:

(1) $$r_{s}\cdot CH_{2.67}O+r_{o}\cdot O_{2}+r_{n}\cdot NH_{3}⟶r_{x}\cdot CH_{1.77}O_{0.49}N_{0.24}+r_{c}\cdot CO_{2}+r_{w}\cdot H_{2}O$$

An elemental biomass composition CH_1.77_O_0.49_N_0.24_ of an *E. coli* K12 strain was used [31,32]. The model was simplified with the assumptions of aerobic growth on a single carbon and energy source, and without product formation. In addition to the mass balances, a degree of reduction (DoR) balance was used. The degree of reduction in the used elements can be defined as follows: $\gamma_{N}=-3$, $\gamma_{C}=4$, $\gamma_{O_{2}}=-4$, $\gamma_{H}=1$ [32]. As this definition leads to a DoR of 0 for NH_3_, $r_{N}$ does not appear in either of the applied balances and can remain undetermined. Hence, the equation system remains over-determined, as there are two balances (C and O) for the calculation of one conversion rate ($r_{X}$). Normalizing all carbon-containing species on a 1 C-mole basis, the following equation system with the respective yield coefficients ($Y_{i,j}$ in mol of i/mol of *j*) was obtained:

(2) $$\mathrm{C-balance}:1+Y_{x,CO_{2}}-Y_{x,gly}=0$$

(3) $$\mathrm{H-balance}:1.77+Y_{x,H_{2}O}\cdot 2-Y_{x,gly}\cdot 2.67-Y_{x,{NH}_{3}}\cdot 3=0$$

(4) $$\mathrm{O-balance}:0.49+Y_{x,{CO}_{2}}\cdot 2+Y_{x,H_{2}O}-Y_{x,gly}-Y_{x,O_{2}}\cdot 2=0$$

(5) $$\mathrm{N-balance}:0.2-Y_{x,{NH}_{3}}=0$$

(6) $$\mathrm{DoR-balance}:4.19+Y_{x,gly}\cdot 4.67-Y_{x,O_{2}}-4=0$$

#### 2.5.2. Oxygen Uptake Rate and Carbon Emission Rate

The oxygen uptake rate (OUR) and carbon emission rate (CER) were calculated from the air flow into the bioreactor $F_{a,in}$, the bioreactor volume $V_{r}$, the molar volume of an ideal gas at 25 °C $V_{m}$, and the gas fractions $y_{i,in/out}$ of $O_{2}$ and $CO_{2}$ in the in- and outflow stream according to Equations (7) and (8). The volumetric fractions of $CO_{2}$ and $O_{2}$ of the inflow gas are assumed constant at $y_{O_{2},in}=0.041$ and $y_{CO_{2},in}=20.91$, respectively. Accumulation of oxygen or carbon dioxide in the fermentation broth can be neglected as their solubility is a function of temperature and pH, which is controlled and assumed constant. Therefore, the OUR and CER are equal to the respective conversion rate $r_{o}$ and $r_{c}$.

(7) $$r_{o}=OUR=\frac{F_{a,in}}{V_{m}\cdot V_{r}}\cdot \left(-y_{O_{2},in}+\frac{1-y_{O_{2},in}-y_{CO_{2},in}}{1-y_{O_{2},out}-y_{CO_{2},out}}\right)\cdot y_{O_{2},out}$$

(8) $$r_{c}=CER=\frac{F_{a,in}}{V_{m}\cdot V_{r}}\cdot \left(-y_{CO_{2},in}+\frac{1-y_{O_{2},in}-y_{CO_{2},in}}{1-y_{O_{2},out}-y_{CO_{2},out}}\right)\cdot y_{CO_{2},out}$$

#### 2.5.3. Biomass and Substrate Conversion Rate

Equations (9) and (10) are derived from the equation system presented in the Equations (1)–(6). Coupled with Equations (7) and (8), the conversion rates for biomass (Equation (9)) and substrate (Equation (10)) can be estimated over the course of the batch process.

(9) $$r_{x}=-8.32\cdot r_{o}-9.71\cdot r_{c}$$

(10) $$r_{s}=-8.32\cdot r_{o}-8.71\cdot r_{c}$$

The C-mole normalized molecular mass of the substrate is $M_{gly}=30.701$ g mol${}^{-1}$ and that of the biomass is $M_{gly}=24.996$ g mol${}^{-1}$. They are used to convert the conversion rates from a C-mole basis to gram. Since the cell specific growth rate $\mu =r_{x}$, the following equation can be formulated for the biomass concentration $c_{x}$.

(11) $$c_{x}=\frac{1}{\mu }\cdot \frac{dc_{x}}{dt}$$

Equation (11) is integrated numerically by trapezoidal rule using the *trapz()* function of the *NumPy* Python library with an initial concentration value of $c_{x,0}=2.5$ g L${}^{-1}$. The specific growth rate μ was calculated within the exponential growth phase. To avoid the impact of the lag phase, only the last 60% of the batch was taken into account when calculating the specific growth rate. A more detailed description of the model is given in the Jupyter notebook provided in the Supplementary Materials.

#### 2.5.4. Cumulative Number of Cell Divisions

Cell concentrations are calculated using the average molecular weight of *E. coli* $m_{E.\;coli}$ with the aforementioned cell composition. The number of cells can be calculated with following relationship:

(12) $$N=\frac{c_{x}\cdot V_{r}}{m_{E.coli}}$$

where $c_{x}$ is the biomass concentration in g L${}^{-1}$, $V_{R}$ is the reactor volume in L, and $m_{E.coli}$ is the dry mass of a single *E. coli* cell in g. The average cell mass of $m_{E.coli}=2.90\times 10^{-13}$ g from the literature was used [33]. The same cell mass was also used for calculation of CCD in the reference literature [25]. The number of generations was calculated per batch *i* according to Equation (13) and used for the calculation of the cumulative number of cell divisions CCD using Equation (14), where $n_{i}$ is the number of generations in batch *i*, $N_{0,i}$ is the number of cells at the start, and $N_{i}$ the number of cells at the point of interest, i.e., the end of the batch. The cumulative number of cell divisions is defined as the sum of the cell divisions of *m* consecutive batches [25].

(13) $$n_{i}=\frac{log\left(N_{i}/N_{0,i}\right)}{log2}$$

(14) $$CCD=\sum_{i=1}^{m}N_{0,i}\left(2^{n_{i}}-1\right)$$

#### 2.5.5. Preprocessing

Process data captured by the parallel bioreactor system and the off-gas analyzer were stored in the InfluxDB and subsequently downloaded as .csv file. The time-series datasets of each bioreactor were separated into the respective batch datasets using a pattern detection algorithm that identifies the process phases as introduced in Figure 2. To smooth out short-term signal fluctuations, a moving average was applied with a window width of n = 6 measurements of 15 s using the *rolling()* function of the Python software library *pandas*. The datasets of the $CO_{2}$ and $O_{2}$ concentrations of each batch were fitted to an exponential function of the type $f\left(x\right)=a\cdot e^{b\cdot x}+c$ using the *optimize.curve_fit()* function of the Python *SciPy* library. Datasets with noisy or irregular process data resulting in a fit with an $R^{2}$ below 0.98 were discarded and not evaluated. A baseline correction was performed to set the y intercept of the fit to the concentration of the inflow of the respective gas for all batches after the first batch to make up for sensor shift over the course of the experiment and for unreliable data within the first 10 min after the reactor content replacement.

## 3. Results

### 3.1. Process Characteristics and Duration

The proposed method was validated by executing ALE growth rate optimization experiments of *E. coli* K12 MG1655 on the non-native carbon source glycerol with the mutagens NTG and a control group without the mutagens NTG and comparing the findings with the results of the reference literature [2,25,26]. Experiments with glycerol and mutagen were named GM1, GM2, and GM3, and experiments with glycerol only were named G1, G2, and G3. The automated ALE experiments were conducted for up to 200 h, resulting in a total of 19–25 sequential batches, after which, they were terminated manually. Between batches, an average reactor volume exchange duration of 42.22±3.86 min was recorded, which was dependent on the maximum possible speed of the respective channels of the peristaltic pumps, which varied between 25–28 mL min${}^{-1}$.

**Figure 3:**
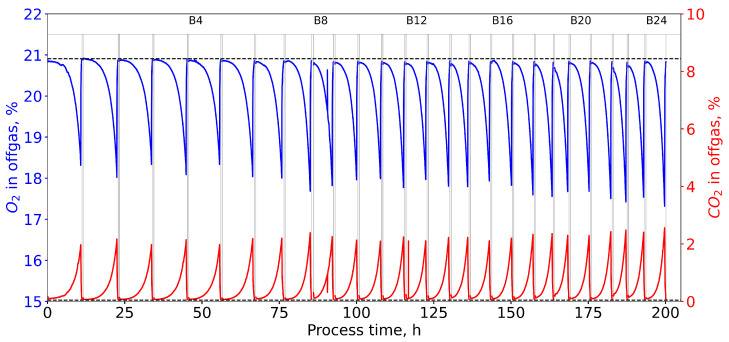
The concentrations of $O_{2}$ and $CO_{2}$ in the off-gas of an automated ALE experiment (GM2) in an L scale stirred-tank bioreactor are shown over the course of 25 consecutive batch experiments totaling a process duration of 200 h. A culture of *E. coli* K12 MG1655 was grown with RB medium at 37 °C, 600–1400 rpm, 40 vvm, and an initial glycerol concentration of 12 g L${}^{-1}$ as sole carbon source. The ALE process was performed in an automated system in a repeated batch mode with a bioreactor volume of 575 mL. Vertical lines indicate the start and end of the medium exchange procedure between batches (grey). The concentrations of $O_{2}=20.91\%$ and $CO_{2}=0.04\%$ in the pressurized air in the inflow are depicted by the horizontal, dashed lines (black). Batch numbers are indicated with B4–B24. The shown data were used as input for the black box model to calculate OUR and CER, as well as derived state variables, such as the estimated biomass and substrate concentrations and the specific growth rate according to Equation (7)–(10).

Figure 3 shows $CO_{2}$ and $O_{2}$ off-gas data of one of the experiments, with NTG highlighting the apparent continuous decrease in the process duration over the course of the experiment. ALE batch duration of *E. coli* cultures without NTG decreased by 40.7% from 11.17 h at batch 2 to 6.62 h at batch 22, whereas, in cultures with NTG, the average batch duration decreased by 45% from 10.29±0.71 h at batch 2 to 5.67±1.31 h at batch 22. The process data are provided in the Supplementary Materials.

### 3.2. Model Predictions of Specific Growth Rate and Biomass Concentrations

**Figure 4:**
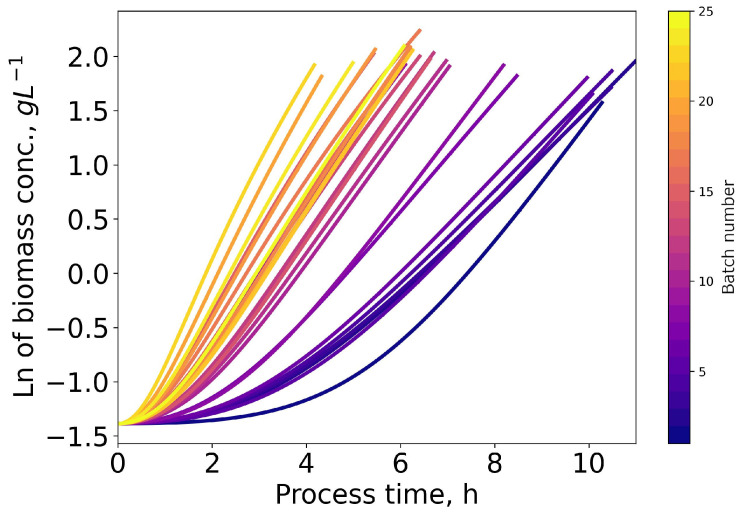
The logarithm of the biomass concentration is depicted over the process time. Each line represents a single batch of the same ALE experiment (GM2). The chronological order of the batches is indicated by the color bar. Early batches show an extended period with a non-linear relationship (lag), which decreases in length as the culture evolves towards a stable phenotype.

Figure 4 shows the logarithm of the estimated biomass concentrations of all batches of the ALE experiment GM2 depicted in Figure 3 as calculated from the black box model introduced in Equations (1)– (6). Early batches experience a long lag and transition phases before entering fully exponential growth. This effect is greatly reduced as cultures evolve towards improved glycerol metabolization and growth. Calculating specific growth rates over the full batch duration would be impacted severely by this effect. Hence, the specific growth rates presented in the following sections were calculated based on the last 50% of the respective batches data, only taking into account the linear slope of the curves.

### 3.3. Comparison of Serial Passaging and Repeated Batch Process

**Figure 5:**
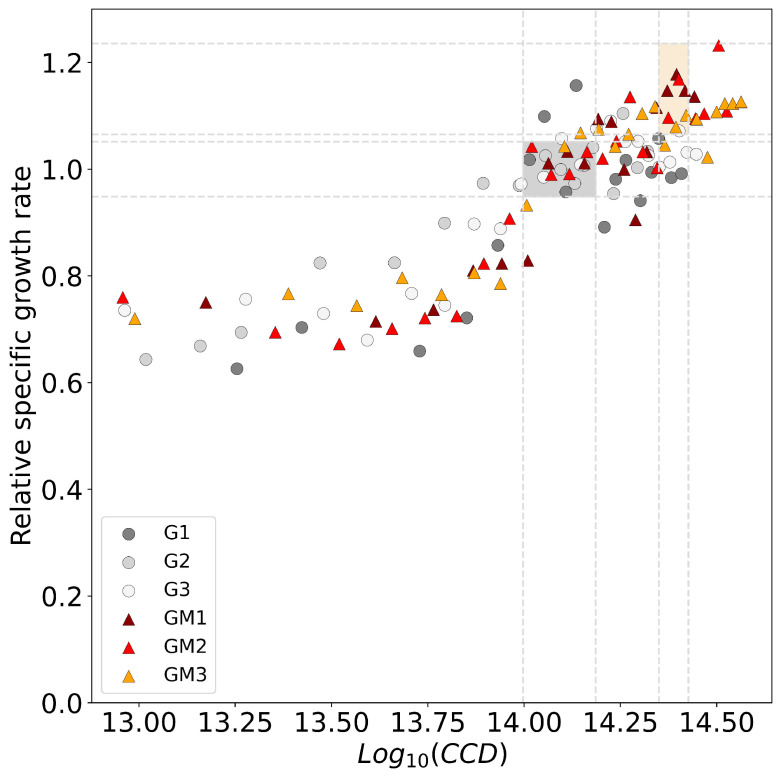
The relative fitness of replicate ALE experiments with *E. coli* K12 MG1655 growing with the non-native carbon source glycerol. Relative fitness is defined as the stable specific growth rate divided by the average stable specific growth rate of the control group of WT *E. coli* without NTG. The cumulative number of cell divisions (CCD) is used as time scale to measure adaptation progress. The specific growth rate is considered stable if the moving average of three consecutive batches has an absolute standard deviation < 0.01 h${}^{-1}$ and no further upwards trend. This definition of a stable phenotype is specific to this set of experiments. A decisive criterion was required to make near-real-time decisions while the experiment was running; hence, a variability based approach was chosen that focuses on the change in optimization metric: the specific growth rate. The specific growth rate of the stable phenotype of the *E. coli* wild-type cultures without NTG is 0.61±0.03 h${}^{-1}$ at a $log_{10}\left(CCD\right)=14.09\pm 0.09$ and is used to compare both groups and calculate the relative fitness (grey shaded area, relative fitness of the WT =1±0.05). The observed average specific growth rate of the WT strain with NTG is 0.70±0.05 h${}^{-1}$ and was reached at a $log_{10}\left(CCD\right)=14.39\pm 0.04$ (orange shaded area, relative fitness of the WT with NTG =1.15±0.08).

Specific growth rate results from the ALE experiments that were estimated using the black-box model were validated with the results from at-line DCW measurements (see Supplementary Materials). DCW measurements of selected batches were taken in 2 h intervals. Figure 5 shows the relative fitness of ALE experiments with and without mutagens plotted against the CCD. The calculated specific growth rates were put into the relation of the wild-type stable phenotype to derive a relative fitness parameter. The average specific growth rate of the strains that evolved in the presence of NTG was 0.70±0.05 h${}^{-1}$ compared to 0.61±0.03 h${}^{-1}$ of the culture without the mutagens, which was used as a reference for the calculation of the relative fitness parameter. The key parameters of Figure 5 are summarized in Table 1 and compared with the reference studies. It was observed that the WT cultures with the native mutation rate (G1, G2, G3) arrive at a stable phenotype faster but exhibit a lower stable specific growth rate compared to cultures that were grown with the additional mutagens NTG (GM1, GM2, GM3). While NTG cultures reached comparable specific growth rates as the control group in a similar timeframe at a $log_{10}\left(CCD\right)=14.1$, the adaption process continued further and reached a stable specific growth rate that was 14.8% higher than that of the control with the native mutation rate.

The results of the stable specific growth rates of both experimental groups are within the margin of error of the published reference experiments [2,25,26]. However, the CCD required to attain the stable phenotype differs greatly between the experiments of this study and the literature reference. Whereas the manual serial passaging method results in a total of $3.9\times 10^{11}$ CCDs, the repeated batch method requires a total CCD of $1.26\times 10^{14}$ in cultures of the WT *E. coli* without NTG. A similar order of magnitude is observed in cultures with NTG. In the reference literature in which a manual system was used, the stable specific growth rates were obtained after a total of 1056 h in cultures without NTG [26]. LaCroix et al. (2017) investigated the effect of different passage sizes (0.001–10%) on growth rate adaptation in their automated system. The presented results (passage size 2–4%) are compared with their results of the group with passage size 1%, which plateaued at a comparable growth rate of 0.60±0.01 h${}^{-1}$ after 456 h.

In the presented work, the overall process duration of the repeated batch experiments to reach the similar specific growth rate result was achieved in 127.3±13.6 h without NTG, and 160.8±5.6 h with NTG cultures, respectively. This equates to a reduction in the process duration by 85–88% if compared to the 44-day-long culturing by serial passaging without NTG by Fong et al. (2005) and a 69–75% reduction compared to the 19 days required with the culturing process in the automated system by LaCroix et al. (2017). However, a comparison with Fong et al. (2005) must be interpreted with the caveat that a culture temperature of 30 °C was used instead of 37 °C as in the presented use case.

## 4. Discussion

Adaptive laboratory evolution experiments are laborious and time-consuming. A few automated ALE systems, operating on the microliter and milliliter scale, have been proposed in the past [10,12,14,16]. These systems are typically based on a microtiter plate format that enables biomass determination by measuring $OD_{600}$ with a plate reader. Systems with reaction vessels on the mL scale are agitated with magnetic stir bars and provide OD measurements either with a respective sensor or via at-line measurements with a liquid handling station and plate reader. ALE systems that operate on the microliter and milliliter scale can be parallelized at a lower cost, but are limited to low biomass densities and restricted with regard to process control [14,15,16]. The lack of pH and DO control or the acquisition of the respective data leads to changing and unknown process conditions that directly impact the stresses exerted on the culture. The proposed system is agitated, aerated, and enables appropriate process control, resulting in transparent and reliable culture conditions. However, the quality of the data acquired could be further improved in the future by using OD probes for biomass determination to obtain an additional source for real-time biomass concentration data.

The successful modeling of batch processes is highly dependent on the quality of the state parameters and the starting conditions as the error is propagated and aggregated over time. Hence, the assumption of a constant biomass concentration at each batch start introduces a small error into the system. Furthermore, it was observed that $CO_{2}$ concentrations in the off-gas at the batch start spiked briefly before going down to expected levels. The effect was observed only in subsequent batches but not the first, and is most likely caused by the stripping of $CO_{2}$ from the fresh medium, whereas the medium of the first batch was already equilibrated during the DO-calibration procedure before the process start. An exponential fit was performed on the data to reduce the impact of this spike. However, going forward, the over-determination of the equation system could be used and further state variables, such as dissolved oxygen or pH, could be added to extend the model with a data reconciliation strategy to reduce the error of the estimate and even quantify the prediction confidence. A similar strategy has been applied successfully in soft sensors for biomass estimation in fed-batch processes [34,35].

The continuous acquisition of process data allows for the calculation of the estimated specific growth rate at any time during the process. As shown in Figure 4, it is problematic to calculate the specific growth rate of early batches over the full duration of the batch, as lag-phases are prominently exhibited in the presented experimental data. Hence, calculating over the full duration instead of the exponential growth phase only will lead to considerably lower specific growth rates. This could explain the deviation between the lower specific growth rates in the early passages reported in the literature and the higher specific growth rates of early batches in the results presented in this work [25,26]. To avoid this, the authors propose using the specific growth rate during the exponential phase only and disregard the behavior during the lag phase for specific growth rate determination.

The CCD has been proposed in the literature as a time scale for measuring evolutionary change in ALE experiments [25]. However, it has been shown that ALE experiments in L scale bioreactors require a CCD that is two orders of magnitude larger than required on the mL scale to yield the same stable growth phenotype. Comparing the automated L scale system with the manual serial passaging approach, it is found that the manual approach requires considerably more passaging events than the automated repeated batch process to yield the same CCD. Thus, although the shake flask process itself is much slower regarding the total experimental time, it is more efficient when considering the number of propagated beneficial mutations per CCD. The optimization target, the heightened stable specific growth rate, depends on the frequency of beneficial mutations and thus the mutation rate in general. Assuming CCD as a transferable measure of evolutionary change between reactor scales, the assumption that the mutation rate behaves similar over time for the same experiment can be inferred. Hence, either the behavior of the mutation rate differs between the compared experimental setups or the deviation between the reported CCDs in the literature and the respective CCDs presented in this work must have another origin.

Campos et al. (2008) showed in their statistical analyses that population bottlenecks influence the population adaption rate [36]. Hence, the starting population size, the imposed bottleneck between batches, has an impact on the speed of evolution. LaCroix et al. (2017) investigated the relationship of the passage size on the adaption rate of the same biological system of *E. coli* K12 MG1655 growing on glycerol and found that increasing and constant passage sizes (0.001–10%) lead to a faster adaption regarding the specific growth rate [16]. In manual experiments, the initial biomass concentration is adjusted for each subsequent passage; hence, passage sizes vary and decrease over time. In the presented experiment, as well as in LaCroix et al. (2017), initial biomass concentrations between batches were kept constant, which contributes to the reduction in the experiment duration.

Mutation rate dynamics in bacterial populations are complex and some sources suggest that mutation rates can increase dramatically in stressful, changing environments in order to counterbalance robustness with evolvability [37,38,39]. Populations in uncontrolled shake flask experiments may be subjected to pH-shift and oxygen limitations. Furthermore, the passaging process as well as a potentially prolonged residence in the stationary growth phase under limited substrate conditions may contribute to heightened stress levels and thus lead to elevated mutation rates and the lower cumulative number of cell divisions required in shake flask experiments. Thus, the controlled process conditions and the proposed drain/fill approach reduce the impact of unwanted selection pressures, including unnoticed fluctuations in process conditions or irregular and lengthy passaging procedures.

An automated adaptive laboratory evolution approach reduces the time required to perform ALE experiments in the presented use case. The time savings can be attributed to multiple sources. The efficient process timing reduces batch phases, which are traditionally spread out artificially over the course of 24 h, to the real time required until the end of the exponential growth phase, instead of prolonging this step by step-by-step reduction in the inoculum size. Consistently larger inoculum sizes result in more cell divisions per unit of time and a reduction in the lag phases [22,23,24]. The automated approach also reduces the time of passaging to approximately 42 min and reduces the amount of manual labor and at-line measurements.

Long-term cultivations can result in biofilm formation. In the context of ALE experiments, biofilm formation would result in an unwanted carry-over of biomass from previous batches. This may favor mutations that contribute to biofilm formation and thus introduce a new selection pressure. The extent of this stress decreases with an increasing reactor size as the relative fraction of passaged biofilm decreases with larger passage size volumes. In the presented use case of the repeated batch process, no visible biofilm formation was observed over the course of 200 h of cultivation. However, further extended cultivation times may eventually result in biofilm formation, especially if heavy frothing is encountered. The use of other microorganisms may result in issues with biofilm formation most likely along the stirrer axis or along the liquid level border. Anti-foam agents could be used to counter biofilm formation and reduce the biofilm build-up.

Many evolution experiments are run for a longer duration than the presented use-case. The maximum duration of the presented method is only dependent on the wear and tear of the bioreactor system. Maintenance during operation is restricted to the exchange of the off-gas air filters every four days to avoid potential pressure build up due to clogging

The current experimental set-up was limited to four parallel stirred-tank bioreactors. However, future improvements of this set-up may include miniaturization and further parallelization. Recent studies have shown the reproducibility and scalability of the used L scale stirred-tank bioreactor to a 48-parallelized-bioreactor system on the mL scale that is integrated into a liquid handling station [40]. With sophisticated bioreactor control software, such a set-up would improve the throughput of ALE experiments greatly and enable automated sampling and at-line sample anaylsis [41,42].

## 5. Conclusions

The authors propose a novel automated adaptive laboratory evolution method that transforms the conventional serial passaging approach from shake flask experiments into an L scale stirred-tank bioreactor that performs serial passaging, in the form sequential batches, automatically. The digitized experimental setup acquires on-line data of all key process and state variables that enable the detection of the end of the exponential growth phase and the online estimation of growth rates using a soft sensor approach. Automated ALE experiments in parallelized stirred-tank bioreactors enable the study of varying mutation rates within one experimental set-up at the same time. This novel ALE method can replace serial passaging experiments to speed up adaptive evolution experiments considerably. With the proposed method, the authors were able to obtain the same stable growth rates of *E. coli* K12 MG1655 with the non-native carbon source glycerol 9.4 times faster than reference serial passaging shake flask experiments and 3.6 times faster than an automated approach on the mL scale. It was further shown that the cumulative number of cell divisions (CCD) is not directly translatable between reactor scales. The cumulative number of cell divisions (CCD) required to obtain the same growth phenotypes as the reference process showed an offset of two orders of magnitude. It is concluded that the time necessary for certain evolutionary progress is not only dependent on the CCD but also dependent on the scale and control of the experiment, as well as the number and size of passages.

## Figures and Tables

**Table 1:** Comparison of the key results of the ALE experiments performed in this study compared to the results from the literature.

Evolution	Method	Replicates	GR (h${}^{-1}$)	CCD	Ref.
WT	mL scale, manual	5 (GA, GB, GC, GD, GE)	0.64±0.04	$3.9\times 10^{11}\pm 0.5\times 10^{11}$	[2,26]
	mL scale, automated	6 (1% passage size)	0.60±0.01	$1.57\times 10^{12}\pm 0.14\times 10^{12}$	[16], suppl.
	L scale, automated	3 (G1, G2, G3)	0.61±0.03	$1.26\times 10^{14}\pm 0.25\times 10^{14}$	this study
WT + NTG	mL scale, manual	2 (GM1, GM2)	0.74	$0.93\times 10^{11}\pm 0.01\times 10^{11}$	[25]
	L scale, automated	3 (GM1, GM2, GM3)	0.70±0.05	$2.45\times 10^{14}\pm 0.21\times 10^{14}$	this study

## Data Availability

The raw process data of the ALE experiments presented in this work, as well as a Jupyter Notebook that contains all data processing and analysis steps, are provided in the Supplementary Materials and on request.

## Supplementary Materials

The following supporting information can be downloaded at: https://www.mdpi.com/article/10.3390/microorganisms11020275/s1.

## Sample Availability

Samples of the final stable phenotype strains are available upon request as cryogenically frozen stock-cultures.

## Abbreviations

The following abbreviations are used in this manuscript:

ALE	Adaptive laboratory evolution
CCD	Cumulative number of cell divisions
CER	Carbon emission rate
DO	Dissolved oxygen
DoR	Degree of reduction
DCW	Dry cell weight
GR	Growth rate
LB	Lysogeny broth
NTG	N-methyl-N’nitro-N-nitrosoguanidine
OUR	Oxygen uptake rate
RB	Riesennberg
SiLA	Standardization in laboratory automation
WT	Wild type
