# Supplementary figures and images for: Accelerated Adaptive Laboratory Evolution by Automated Repeated Batch Processes in Parallelized Bioreactors
Source: Microorganisms. 2023 Jan 20;11(2):275. doi: 10.3390/microorganisms11020275 (PMC9965177; doi:10.3390/microorganisms11020275)

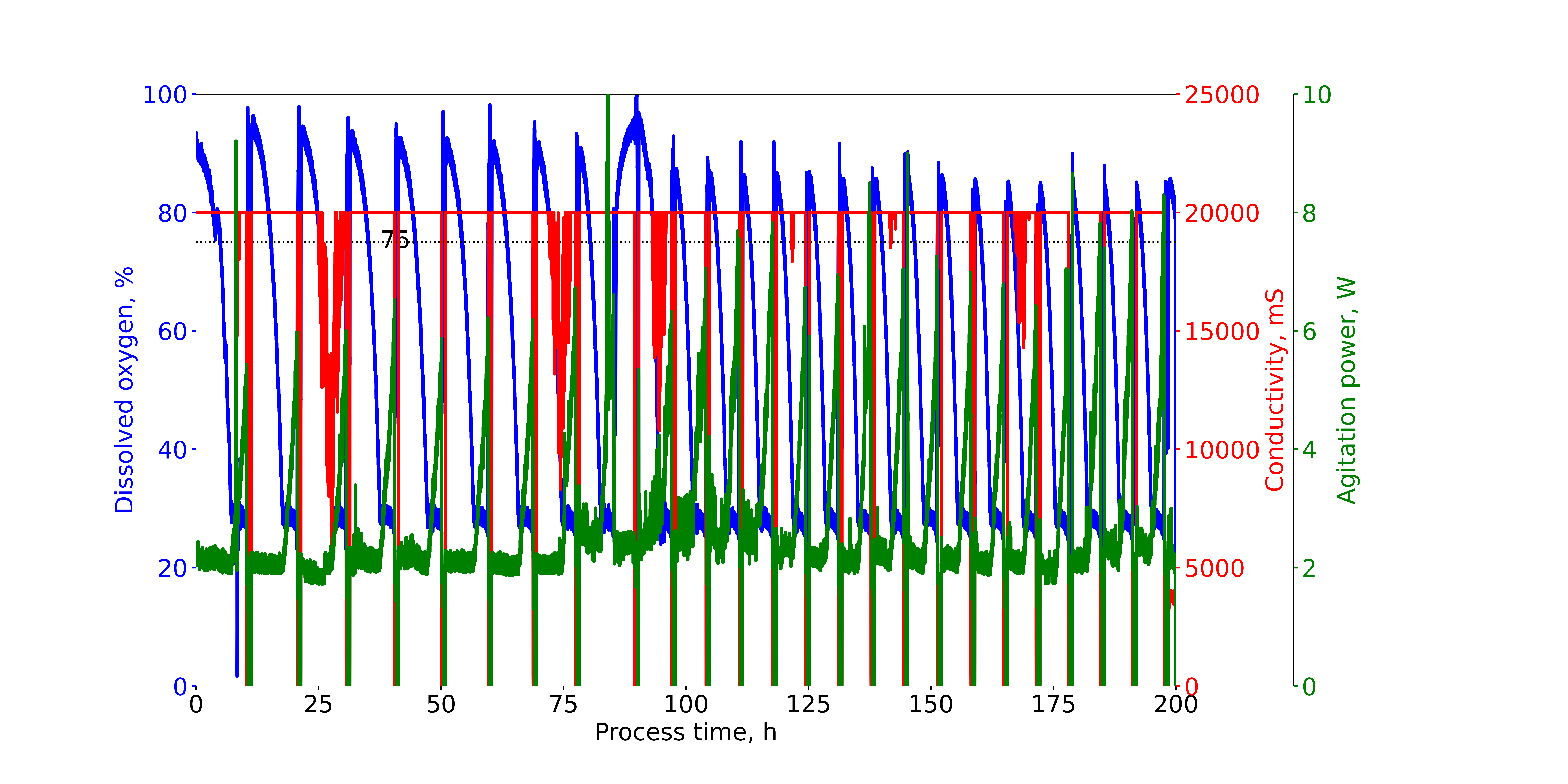

Supplement: Supplementary file 1 [file microorganisms-11-00275-s001.zip › jupyter_supplement/process control/process_control_full.png]

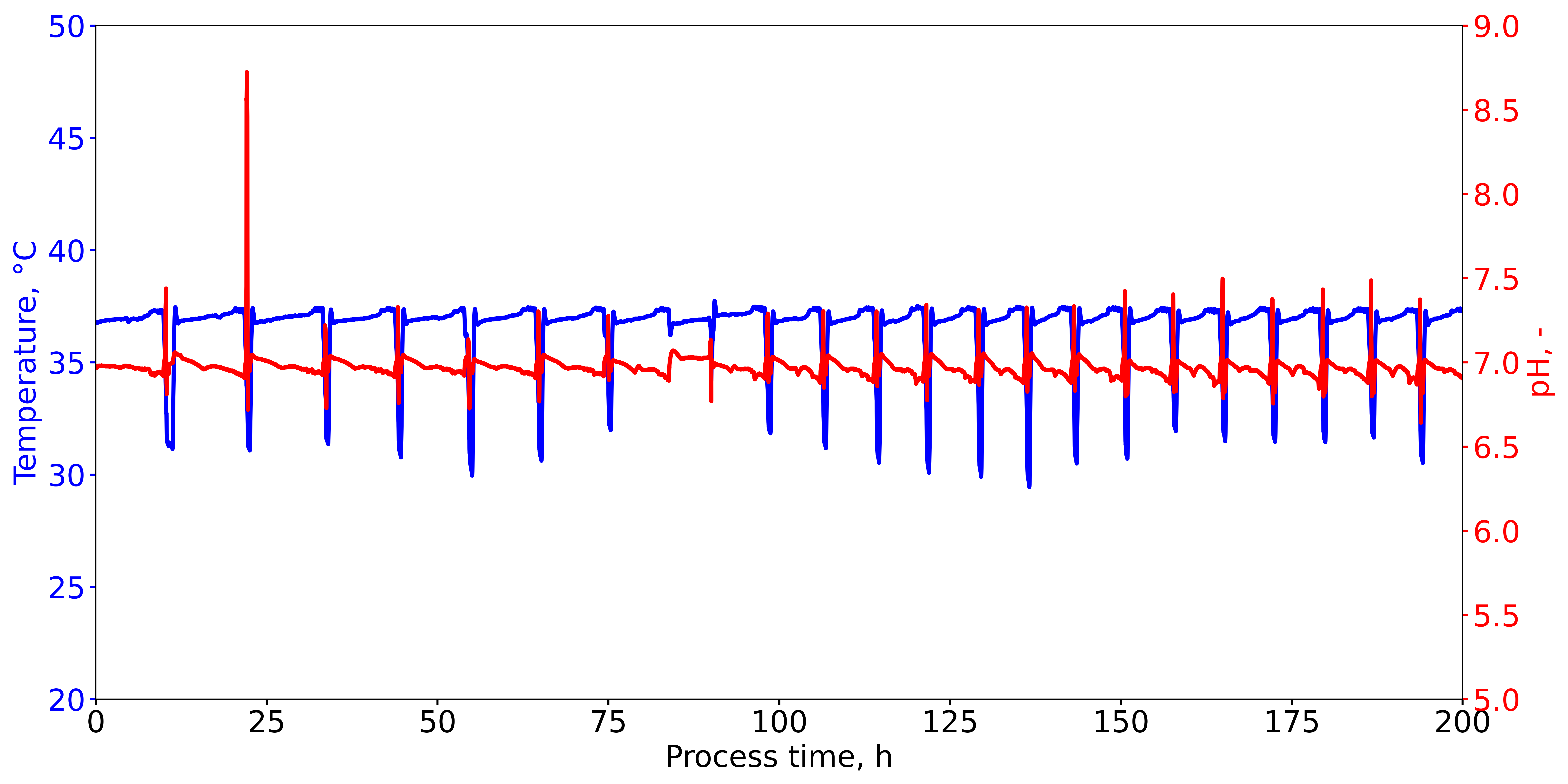

Supplement: Supplementary file 1 [file microorganisms-11-00275-s001.zip › jupyter_supplement/process control/process_control_full_T_and_PH.png]

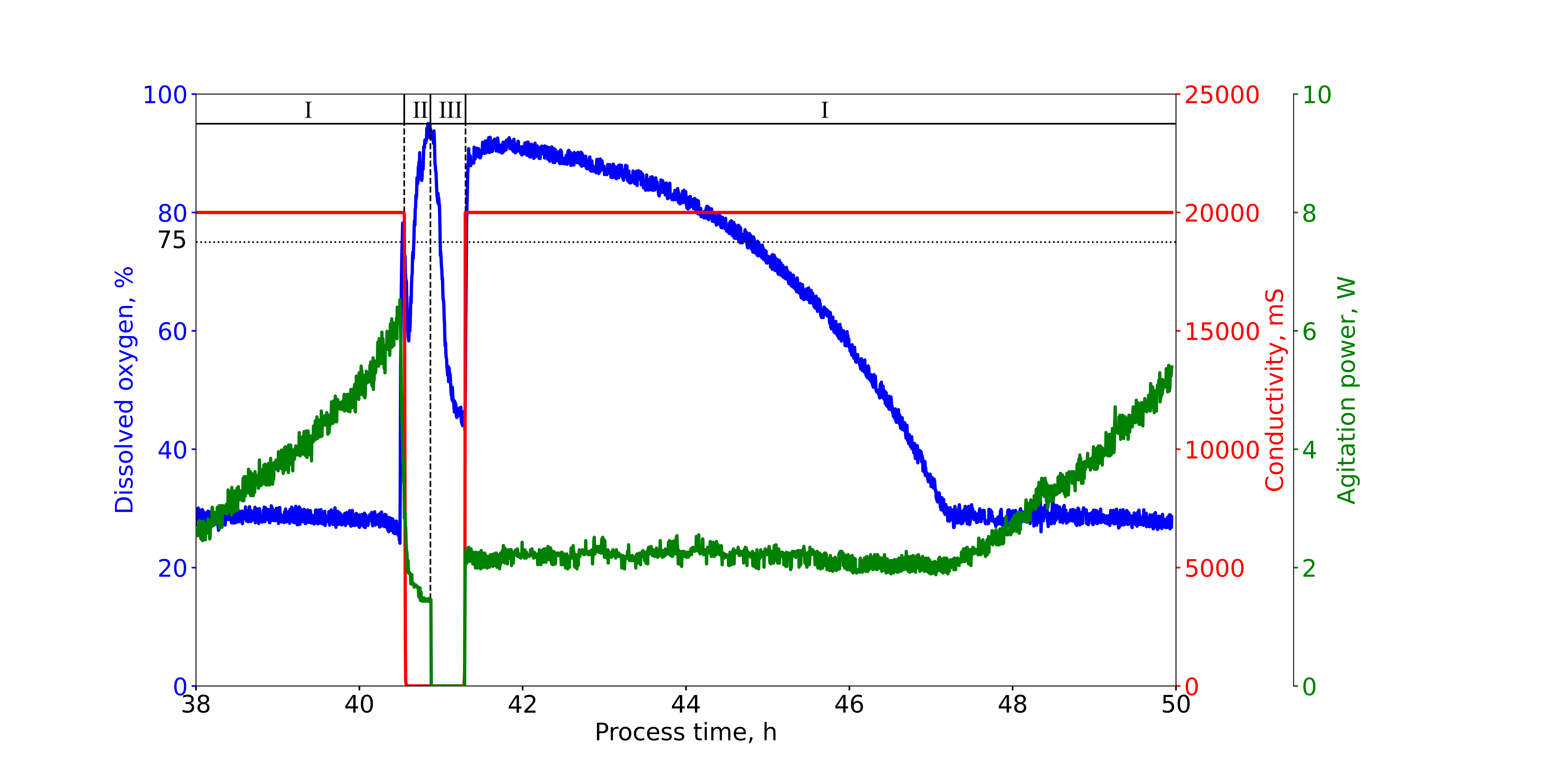

Supplement: Supplementary file 1 [file microorganisms-11-00275-s001.zip › jupyter_supplement/process control/process_control.png]

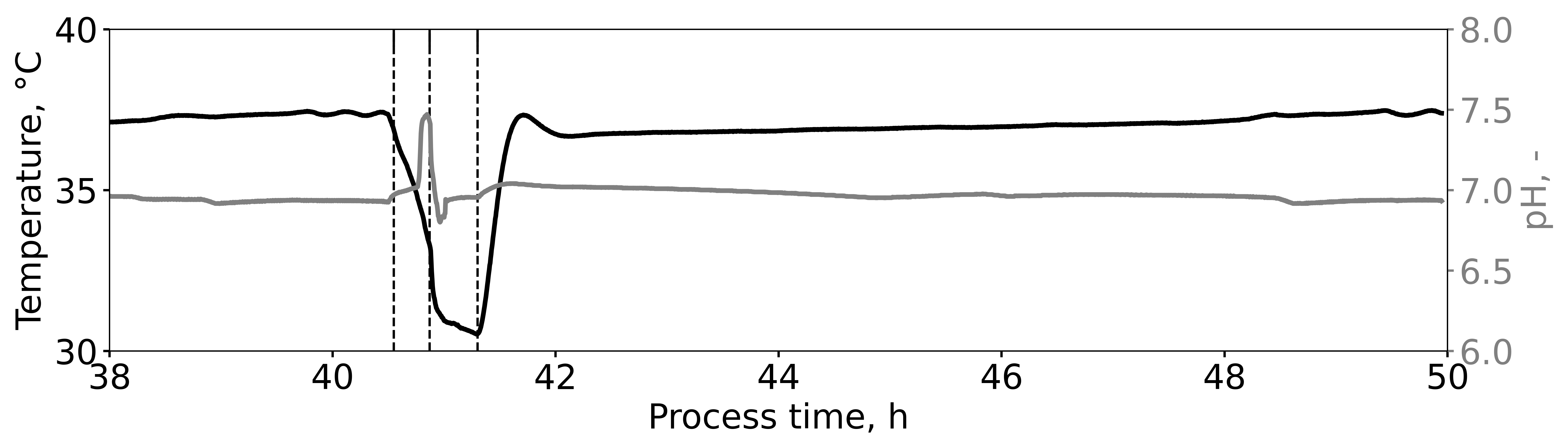

Supplement: Supplementary file 1 [file microorganisms-11-00275-s001.zip › jupyter_supplement/process control/process_control_T_and_PH.png]

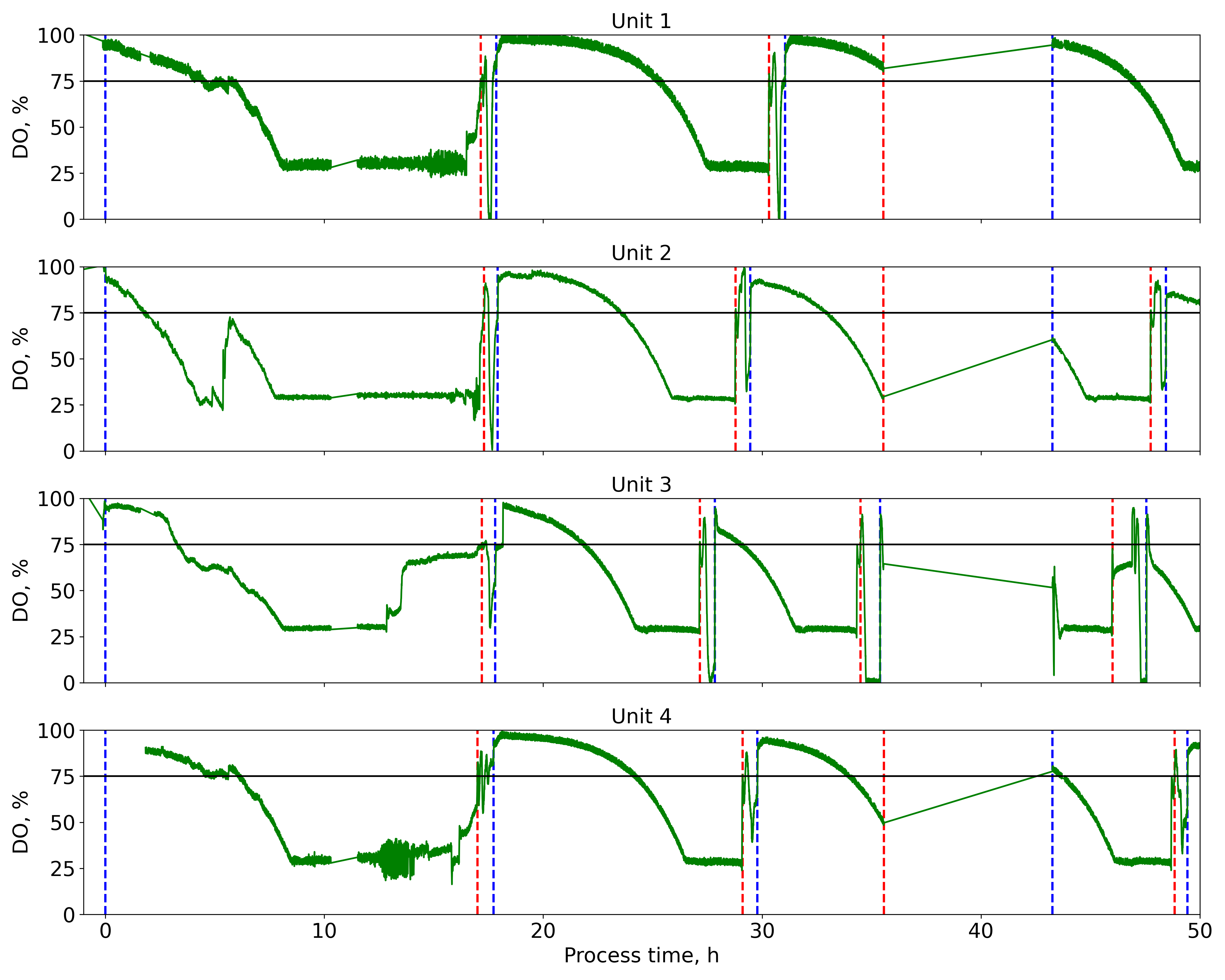

Supplement: Supplementary file 1 [file microorganisms-11-00275-s001.zip › jupyter_supplement/analysis/raw_data/ALE_V_5/Full_process_view.png]

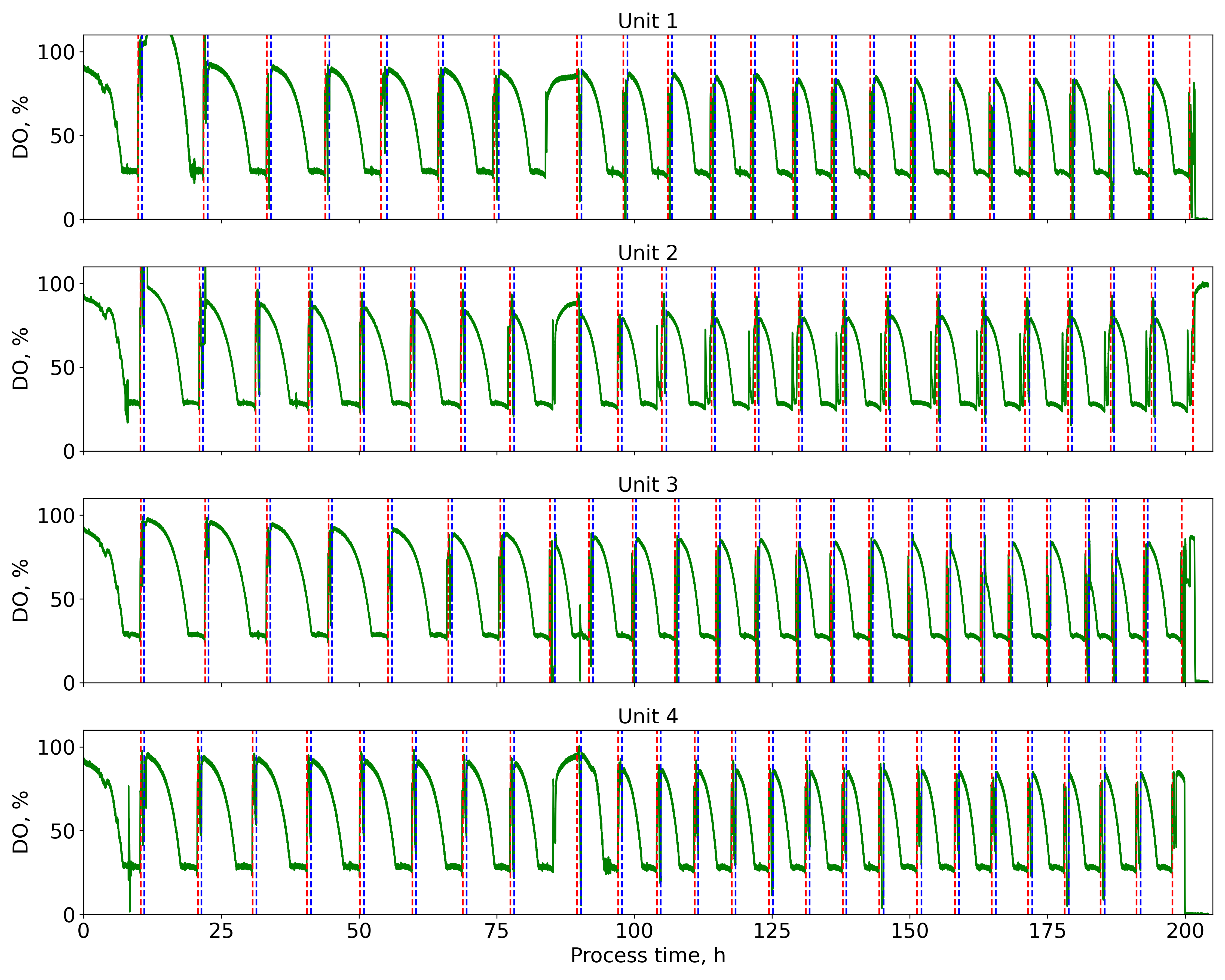

Supplement: Supplementary file 1 [file microorganisms-11-00275-s001.zip › jupyter_supplement/analysis/raw_data/ALE_V_6/Full_process_view.png]
